# Distinct pathophysiological mechanisms of *Heterometrus laoticus* and *Lychas mucronatus* scorpion venoms on cardiovascular and renal functions

**DOI:** 10.1590/1678-9199-JVATITD-2025-0080

**Published:** 2026-06-12

**Authors:** Narongsak Chaiyabutr, Lawan Chanhome, Panithi Laoungbua, Taksa Vasaruchapong, Orawan Khow, Onrapak Reamtong, Asada Leelahavanichkul, Visith Sitprija

**Affiliations:** 1 Queen Saovabha Memorial Institute, Thai Red Cross Society, Bangkok, Thailand.; 2 Snake Farm, Queen Saovabha Memorial Institute, Thai Red Cross Society, Bangkok, Thailand.; 3 Department of Research and Development, Queen Saovabha Memorial Institute, Thai Red Cross Society, Bangkok, Thailand.; 4 Department of Molecular Tropical Medicine and Genetics, Faculty of Tropical Medicine, Mahidol University, Ratchathewi, Bangkok, Thailand.; 5 Department of Microbiology, Faculty of Medicine, Chulalongkorn University, Bangkok, Thailand.

**Keywords:** Heterometrus laoticus, Lychas mucronatus, Scorpion venom, Phospholipase A_2_, Neurotoxin, Cardiovascular, Renal parameters

## Abstract

**Background:**

*Heterometrus laoticus* and *Lychas mucronatus* are widely distributed in Southeast Asia, yet their pathophysiological effects of both venoms remain poorly characterized due to low human fatality rates. This study compared their venom compositions and acute cardiovascular and renal effects.

**Methods:**

Anesthetized male New Zealand White rabbits were monitored for blood pressure (BP), heart rate (HR), and renal clearance following intravenous administration of crude venom (0.5 mg/kg). Venom components were identified via LC-MS/MS, and hematological/biochemical parameters were assessed.

**Results:**

*H. laoticus* venom induced a rapid, transient hypotension (*p* < 0.05), followed by a mild, prolonged hypotensive phase (up to 120 min). Conversely, *L. mucronatus* venom elicited a biphasic response: initial transient hypotension followed by significant hypertension (*p* < 0.05) and a subsequent terminal hypotensive stage. Renal hemodynamic changes in both groups were secondary to these systemic cardiovascular fluctuations.

**Conclusions:**

LC-MS/MS revealed that the neurotoxin-rich profile (KTx and NaTx) of *L. mucronatus* drives vasoconstriction and hypertension. In contrast, the higher PLA₂ content in *H. laoticus* mediates cytotoxic-like effects, resulting in vasodilation and hypotension. These distinct molecular mechanisms suggest that clinical management should be species-specific, even for venoms traditionally considered “mild.”

## Background

Scorpions are arthropods belonging to the class Arachnida and the order Scorpiones, with more than 2,200 described species worldwide [1]. In Southeast Asia, the genera *Heterometrus* and *Lychas* (family Scorpionidae) are widely distributed. *Heterometrus laoticus* and *Lychas mucronatus* typically inhabit humid, forested areas across the Indochinese Peninsula and are commonly found in northern and northeastern Thailand [2]. Scorpion envenomation is widespread; however, envenomation by these species is usually mildly symptomatic and managed with supportive treatment, often producing local pain, inflammation, edema, swelling, and redness at the sting site, which may persist for several hours to a few days [3]. Standard therapeutic approaches for scorpion stings focus primarily on symptomatic relief and supportive care [4]. Current management strategies include the administration of analgesics (such as NSAIDs or local anesthetics like lidocaine), antihistamines, and corticosteroids to address local pain and hypersensitivity [5]. In cases of severe systemic envenomation, management involves intensive care monitoring and pharmacological agents to stabilize hemodynamics. For instance, alpha-blockers like prazosin have emerged as a significant advancement in treating the sympathetic storm and hypertension associated with many scorpion species [6]. However, a major limitation in the clinical management of *H. laoticus* and *L. mucronatus* stings is the lack of species-specific antivenoms. Since no human fatalities have been officially reported for these species, their venoms have not been systematically characterized, often leading to a reliance on generic supportive treatments that may not address specific pathophysiological changes. In contrast to these clinacally “mild” species, stings from other scorpions can cause systemic manifestations, such as cardiorespiratory distress syndrome and nephropathy, which are rare and have been reported mainly in the Middle East and North Africa. Stings from certain other scorpion species, for example, *Mesobuthus tamulus*, *Tityus discrepans*, and *Tityus serrulatus*, have been reported to cause severe envenomation and high mortality, particularly among children. Such cases are primarily associated with cardiorespiratory complications characteristic of scorpion envenomation syndrome. Cardiovascular manifestations typically occur in two distinct phases: an initial hyperdynamic phase, characterized by hypertension, tachycardia, and increased myocardial contractility, followed by a hypokinetic phase marked by hypotension and impaired left ventricular systolic function [7, 8, 9].

Scorpion venoms are complex mixtures of polypeptides with diverse biological activities. For example, approximately 200 toxin components have been identified in the venom of *Heterometrus petersii*, a species closely related to *H. laoticus* [10]. However, it remains unknown whether the toxin components present in the venom of *H. laoticus* or *L. mucronatus* induce physiological changes following envenomation. In the present study, we analyzed the protein profiles of *H. laoticus* and *L. mucronatus* venoms using LC-MS/MS. Various peptides, proteins, and atypical toxin families were identified, revealing alterations in systemic physiological responses post-envenomation associated with the distinct composition of each venom, and suggesting the presence of novel biologically active polypeptides.

Based on this information, the present study was designed to evaluate whether the venoms of *H. laoticus* and *L. mucronatus* collected in Thailand induce severe systemic cardiovascular or renal toxicity. Although only a few studies have documented their effects, both venoms are generally known to cause intense local pain, swelling, erythema, and transient disturbances in blood pressure. To address this issue, we investigated the extent to which intravenous administration of sublethal doses of *H. laoticus* or *L. mucronatus* venoms is associated with time-dependent changes in cardiovascular and renal functions throughout the experimental period. Additionally, hematological and biochemical parameters were assessed to provide further insights into the pathophysiological alterations induced by scorpion envenomation.

## Methods

### Animals

Adult male New Zealand White rabbits (2-3 kg) were obtained from the Animal House, Queen Saovabha Memorial Institute (QSMI), and housed under controlled conditions (standard diet, water *ad libitum*, 12-h light/dark cycle, 26 ± 1 °C). Animals were acclimatized for two weeks before experiments. All procedures complied with the National Research Council of Thailand guidelines and were approved by the QSMI Animal Care and Use Committee (QSMI-ACUC-08-2024). 

### Scorpions and venom collection

Venom was obtained from approximately 250 adult *H. laoticus* and 200 adult *L. mucronatus* scorpions collected in the northeastern and southern regions of Thailand. The scorpions were maintained at the Snake Farm, Queen Saovabha Memorial Institute, Thai Red Cross Society, and were fed crickets and provided with water *ad libitum*. Venom extraction was performed by electrical stimulation using a stimulator (Grass SD9, USA) with the following parameters: frequency 12 pps, pulse duration 10 ms, and 12-14 V DC. The crude venom was recovered by mixing it with a small volume of distilled water, followed by centrifugation at 5,000 rpm for 10 minutes. The resulting supernatant was lyophilized and stored at −20 °C until use.

### LD_50_ determination for *H. laoticus* and *L. mucronatus* venoms

The median lethal dose (LD_50_) of crude venom was determined in ICR mice (*Mus musculus*, 18-20 g body weight) following intravenous injection via the tail vein. Lethal toxicity was assessed by intravenous injection of 0.2 mL of serial 1.2-fold dilutions of lyophilized venom prepared in sterile 0.9% NaCl solution into the tail vein of mice. Twenty-five mice were divided into five groups (n = 5 per group) and administered increasing doses of either crude *H. laoticus* or *L. mucronatus* venom. All mice were observed for 24 h for mortality. The cumulative numbers of surviving and dead mice at each venom dose were recorded after 24 h of venom injection. The LD₅₀ was calculated from the percent mortality data. The LD₅₀ and its 95% confidence limits were calculated using the arithmetical method of Reed and Muench [11], according to WHO guidelines. LD₅₀ determination was based on log doses, numbers of dead and surviving mice, cumulative deaths, cumulative survivors, cumulative totals, mortality rate, percent survival, and percent mortality, as calculated by the following formula:

$$\log{LD}_{50}=\log\;\left(\mathrm{lower dilution}\right)+\left[\log\left(\mathrm{dilution factor}\right)\;\times \;\frac{\left(50-\mathrm{\% mortality at lower dose}\right)}{\left(\mathrm{\% mortality at upper dose}-\mathrm{lower dose}\right)}\right]$$

The calculated LD₅₀ values were 22.2 mg/kg for H. laoticus venom and 14.8 mg/kg for L. mucronatus venom.

### Animal preparation

An experimental study was conducted to evaluate the effects of systemic scorpion venom administration on cardiovascular and renal functions over 120 minutes. Adult male rabbits were anesthetized with pentobarbital sodium (25 mg/kg, i.v.) and catheterized with polyethylene tubing. The left marginal ear vein was cannulated for venom administration and fluid infusion during renal clearance studies. A heparinized cannula (18-G) was inserted into the right central ear artery for continuous monitoring of arterial blood pressure and heart rate using a force-displacement transducer connected to a physiograph (Polygraph, Grass Model 79), as well as for collecting blood samples at different time intervals after venom injection for hematological and biochemical analyses. Arterial blood samples were collected at 0, 5, 15, 30, 60, 90, and 120 minutes after venom injection for hematological and biochemical analyses. For renal function assessment, urine was collected via a polyvinyl urethral catheter inserted into the urinary bladder to measure the urine flow rate at different time intervals after venom injection. 

### Experimental design

*Venom dose optimization*

The dose range was selected based on preliminary dose-response experiments to identify concentrations that produced measurable biological effects with minimal cytotoxicity. Pilot studies revealed that the dose of lyophilized H. laoticus or L. mucronatus venom that caused minimal cytotoxicity in rabbits, was 0.5 mg/kg body weight (BW) after intravenous injection, although the LD_50_ values were calculated as 22.2 mg/kg for H. laoticus venom and 14.8 mg/kg for L. mucronatus venom. This study aims to provide a compromise that optimizes the combination of renal and cardiovascular function, while also achieving a survival time of at least 3 hours. Moreover, the i.v. administration of venom at 0.5 mg/kg BW produced minimal hemodynamic alterations, whereas at higher doses, the marked changes in general circulation were progressive, leading to cardiovascular collapse and, in some instances, rapid death.

*Determination of cardiovascular and renal function*

Following animal preparation, an intravenous priming dose of 0.9% NaCl containing inulin (25 mg/kg) and para-aminohippuric acid (PAH, 6 mg/kg) was administered, followed by a continuous infusion of 0.9% NaCl containing inulin (500 mg%) and PAH (120 mg%) at 1.0 mL/min throughout the experiment. After a 30-minute equilibration period (control), measurements of blood pressure, heart rate, hematological parameters, and renal clearance were initiated prior to the intravenous administration of scorpion venom (H. laoticus or L. mucronatus). Urine was collected every 10 minutes, with midpoint arterial blood samples taken simultaneously. Effective renal plasma flow (ERPF) and glomerular filtration rate (GFR) were determined from PAH and inulin clearance, respectively [12, 13]. Renal clearance was assessed during the control period and at 5, 15, 30, 60, 90, and 120 minutes after venom injection. At each time point, 0.5 mL of arterial blood was collected into heparinized tubes and a urine sample was collected for renal function and biochemical assays.

*Calculation of renal functions*

Renal clearance (C) was calculated as C = UV/P, where U is the urine concentration, V is the rate of urine flow (UF), and P is the plasma concentration] using the plasma and urine inulin and PAH levels for each period. Inulin clearance (C_in_) was used to estimate the glomerular filtration rate (GFR), while the PAH clearance (C_PAH_) was employed to estimate the effective renal plasma flow (ERPF). Effective renal blood flow (RBF) was calculated as follows:

$$RBF=ERPF\;\times \;\frac{100}{100-PCV}$$

Filtration fraction (FF) was calculated as:

$$FF=GFR\;\times \;\frac{100}{ERPF}$$

Osmolar clearance (C_osm_) was calculated as follows:

$$C_{osm}=\frac{U_{osm}\times \;V}{P_{osm}}$$

The fractional excretions of sodium (FE_Na+_), potassium (FE_K+_), and chloride (FE_Cl-_) were calculated as C_Na+_/C_in_, C_K+_/C_in_, and C_Cl-_ /C_in_, respectively. Free water clearance (C_H2O_) was calculated as follows:

$$C_{H2O}=UF-\;C_{osm}$$

Mean arterial blood pressure (MBP) was calculated as follows:

$$MBP=\;\frac{\mathrm{Systolic pressure}+(2\;\times \mathrm{Diastolic pressure})}{3}$$

### Determination of hematological and electrolyte parameters

At each study period after envenomation, 0.5 mL of arterial blood was collected into K₃EDTA tubes for hematological analysis. Parameters measured included red blood cells (RBC), hemoglobin (Hb), mean corpuscular volume (MCV), leukocytes, platelets, neutrophils, lymphocytes, and monocytes, using an automated hematology analyzer (IDEXX ProCyte Dx, Westbrook, USA). In heparinized plasma and urine samples, the concentrations of Na^+^ and K^+^ ions were determined by a flame photometer (Flame Photometers, Laboratory Instrument, BWB Technologies UK Ltd.) and the Cl^-^ ion concentration by a chloridometer (Chloride Analyzer 925, Corning Ltd.). The osmolality was measured using an osmometer (Fiske Micro-osmometer Model 210, Fiske Associates, Norwood, Massachusetts, USA).

### Determination of biochemical parameters of kidney functions

Blood biochemistries, including plasma creatinine and blood urea nitrogen (BUN), were measured with a Vet Test Chemistry Analyzer (IDEXX, UK). These parameters were estimated to evaluate kidney function.

### Venom protein analysis by SDS-PAGE

Venom proteins from H. laoticus and L. mucronatus were analyzed by two-dimensional gel electrophoresis (2D-GE) as described by Berkelman and Stenstedt [14]. For the first dimension, isoelectric focusing (IEF) was performed using 150 µg of venom protein diluted in 125 µL of 60 mM DTT, 4% (w/v) CHAPS, and 0.5% (v/v) immobilized pH gradient (IPG) buffer, loaded onto 7-cm IPG gel strips with a linear pH range of 3-10 (Amersham Bioscience Inc.). Electrofocusing was carried out at 30 kVh using an IPG system at 20 °C according to the manufacturer’s instructions. After IEF, the IPG strips were transferred to the second dimension and separated by SDS-PAGE on 12% polyacrylamide gels. Protein spots were visualized by Coomassie Brilliant Blue R-250 staining.

### Venom sample preparation for proteomics

The protein profiles of each pooled lyophilized venom from adult H. laoticus and L. mucronatus were used in this study. Sample preparation for proteomics analysis was performed using 1 mg of each lyophilized venom, which was lysed in 0.2 mL of lysis buffer containing 0.2% RapiGest SF surfactant (Milford, MA, USA), 10 mM NaCl, and 10 mM ammonium bicarbonate. The lysate was then centrifuged at 12,000 g for 20 minutes at 16 °C, and the supernatant containing proteins was collected, while debris was discarded. Protein concentrations were determined using a BCA assay kit (Pierce, Thermo Fisher, USA), and 50 µg of protein was aliquoted for subsequent processing. Subsequently, 2.5 mM TCEP was added to the samples, which were incubated at 90°C for 15 minutes. After cooling, 12.5 mM IAA was added, and the samples were incubated in the dark at room temperature for 35 minutes. Samples were then cleaned using a desalting column and Zeba spin column, with the flow-through fraction collected. To the cleaned samples, 0.2% RapiGest SF was added at a 1:1 (v/v) ratio, followed by the addition of trypsin (100 ng/µL in ammonium bicarbonate) at a 1:50 (w/w) ratio. The samples were incubated at 37 °C for 6 hours to facilitate digestion. Finally, the reaction was terminated by adding 1% formic acid at a 1:10 v/v ratio. The tryptic peptides were lyophilized and stored prior to LC-MS/MS analysis.

### Venom protein analysis by liquid chromatography with tandem mass spectrometry (LC-MS/MS)

The LC-MS/MS spectrum data were collected in the positive mode with an Orbitrap HF mass spectrometer combined with a nano-LC system equipped with an EasySpray C18 column (Thermo Scientific™ ES903; 75 (m × 50 cm, 2.0 µm). Briefly, mobile phase A consisted of 0.1% formic acid in water, and mobile phase B consisted of 100% acetonitrile with 0.1% formic acid. Separation was conducted with a linear gradient of 5-45% mobile phase B at a constant flow rate of 300 nL/min for 135 min. The tryptic peptides were analyzed by applying a data-dependent acquisition method, followed by higher-energy collisional dissociation. Full scan mass spectra were acquired at an m/z ratio of 400 to 1600 with an AGC target set at 3 ×10^6^ ions, a resolution of 120,000, and an injection time of 20 ms. MS/MS scanning was initiated when the automatic gain control target reached 3 x 10^6^ ions, at a resolution of 15,000, and an injection time of 10 ms. The isolation window was 1.6 m/z. Xcalibur software (Thermo Fisher Scientific) was utilized to automatically collect the mass spectra. Raw LC-MS/MS files underwent analysis using the Proteome Discoverer with the SEQUEST™ HT algorithm (Thermo Fisher Scientific), referencing the UniProtKB (taxonomy: Arachnida, retrieved Dec. 20, 2024, 1,491,248 sequences) adhering to specific criteria: strict trypsin specificity, up to two missed cleavages, a fixed carbamidomethyl modification on cysteine ([C]; +57.0215 Da) and variable modification on methionine ([M]; +15.9949). The relative protein abundance was standardized using the software’s normalization algorithm.

Statistical analysis

The data are presented as mean ± SEM. Statistical analyses were performed using Prism 5.0 (GraphPad Software, San Diego, CA, USA). The differences between the internal control and each specified time point within each experimental group were analyzed using one-way repeated measures ANOVA followed by Bonferroni’s post hoc test, where appropriate. The level of significance was accepted at p < 0.05. 

## Results

### Two-dimensional gel electrophoresis comparison of H. laoticus and L. mucronatus venoms 

**Figure 1.**
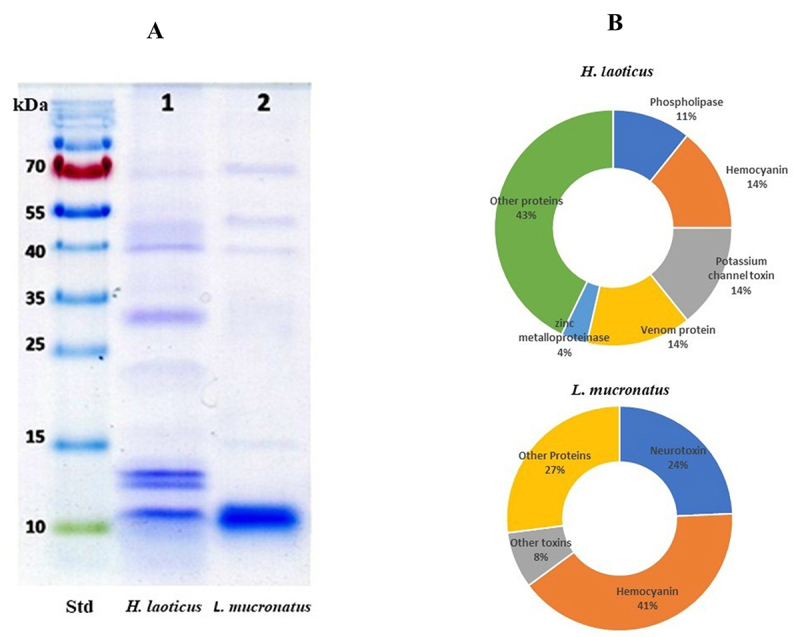
of H. laoticus and L. mucronatus venoms. **(A)** Representative SDS-PAGE gel (30 µg total protein per lane) depicting protein banding patterns of H. laoticus (lane 1) and L. mucronatus (lane 2) venoms. **(B)** Comparative proteomic profiles indicating the relative abundance (%) and distribution of toxin families and specialized components in each venom.

The two-dimensional gel electrophoresis (2D-GE) images (Figure 1A) illustrate distinct differences in venom protein composition between the two scorpion species. H. laoticus venom displays a higher number and intensity of protein spots, particularly in the phospholipase A₂ (PLA₂) region, which was absent in L. mucronatus. In contrast, L. mucronatus venom contained neurotoxin protein spots (~10.8 kDa) which were prominent in L. mucronatus venom but not detected in H. laoticus. Proteins below 10 kDa were not resolved in this analysis. These differences highlight species-specific venom profiles based on molecular mass and isoelectric point (pI).

### Comparative distribution of venom protein categories in H. laoticus and L. mucronatus 

The comparative proteomic profiles of H. laoticus and L. mucronatus venoms reveal distinct patterns in toxin composition and specialization (Figure 1B.). In H. laoticus, the majority of venom components are classified as other proteins (43%), followed by hemocyanin (14%), venom proteins (14%), potassium channel toxins (14%), phospholipases (11%), and zinc metalloproteinase (4%). This distribution suggests a relatively diverse but less specialized venom containing both enzymatic and structural proteins that contribute to general cytotoxicity and tissue damage rather than targeted neurotoxic effects. In contrast, L. mucronatus venom is dominated by hemocyanin (41%) and a high proportion of neurotoxins (24%), alongside other proteins (27%) and a smaller fraction of other toxins (8%). The presence of a larger neurotoxic component indicates that L. mucronatus venom is more specialized and potent in targeting the nervous system, consistent with its more rapid paralytic action. Overall, while H. laoticus exhibits a broad-spectrum venom profile with enzymatic activity, L. mucronatus shows a clear neurotoxic specialization that enhances its predatory and defensive efficiency.

### Proteomic profiles of H. laoticus and L. mucronatus venoms 

The protein profiles of H. laoticus and L. mucronatus venoms were analyzed using LC-MS/MS. Comparative proteomic analysis revealed that 28 proteins were upregulated in H. laoticus venom (Table 1). Among these, the most significantly upregulated were phospholipase A₂ phospholipin (P0DKU2), 5′-nucleotidase (A0A1W7RA44), and potassium channel toxin KTx 4.1 (P0DJ40). Several hemocyanin subunits were also found to be upregulated. Notably, potassium channel toxin κ-KTx 4.1 (P0DJ40) and venom toxin (A0A1L4BJ71) were identified as the dominant toxins in H. laoticus venom. In contrast, 37 proteins were upregulated in L. mucronatus venom (Table 2), with the most prominent including neurotoxin β-KTx 31.1 (P0CI49), an uncharacterized protein (A0AAV6TWV6), and β-toxin BmKAs1 (Q9UAC8). Additionally, multiple isoforms of neurotoxins and β-toxins were identified as the major components of L. mucronatus venom, in contrast to those of H. laoticus.

**Table 1.** The most upregulated venom proteins (ranked by Sequest HT score) identified in H. laoticus venom. Data derived from LC-MS/MS analysis of the tryptic peptides.

No	Accession	Description	Coverage [%]	# Peptides	MW [kDa]	calc. pI	Score sequest HT	Abundance ratio: *(L. mucronatus)/(H. laoticus)*
1	P0DKU2	Phospholipase A_2_ phospholipin	14	2	16.4	7.42	112.09	0.01
2	A0A1W7RA44	5'-nucleotidase	3	1	64.9	5.83	96.81	0.01
3	P0DJ40	Potassium channel toxin kappa-KTx 4.1	16	1	7.5	7.20	76.33	0.01
4	A0AAV4R105	Hemocyanin F chain	9	4	71.5	6.62	70.73	0.01
5	G8YZS4	Hemocyanin subunit g	5	3	72	6.28	68.53	0.01
6	A0A1L4BJ71	Venom toxin	3	1	45.5	6.15	68.03	0.01
7	A0A1W7RAC4	Heat shock 70 kDa protein	18	9	70.9	5.68	63.36	0.01
8	Q9NFL4	Hemocyanin G chain	5	4	71.8	6.18	59.10	0.01
9	A0A1B3IJ23	Venom peptide HtfTx1	12	2	12.7	7.56	52.54	0.01
10	P84094	Potassium channel toxin alpha-KTx 6.13	24	1	3.7	8.51	47.57	0.01
11	H2CYP4	Phospholipase-like calcium release channel inhibitor	14	3	18.6	5.36	31.28	0.01
12	A0A1W7RAI2	Fatty acid-binding protein	16	2	14.5	5.21	26.77	0.01
13	A0AAV1YTX6	Uncharacterized protein (Fragment)	7	2	38.8	5.99	21.28	0.01
14	P0C2F4	Heteroscorpine-1	34	2	10.2	8.56	1553.10	0.01
15	P0DMI6	Phospholipase A_2_ heteromtoxin	51	6	18.2	7.77	2431.33	0.02
16	P59867	Potassium channel toxin alpha-KTx 6.3	26	2	3.8	8.69	85.31	0.02
17	A0A087TQT5	Putative zinc metalloproteinase (Fragment)	12	1	12.1	9.26	29.24	0.02
18	A0AAQ4FFI2	Prohormone-4	6	1	19.8	5.39	23.68	0.02
19	A0A0C4G5K0	Heterin-2	26	2	8.4	9.36	104.75	0.03
20	A0A1W7RA03	Peptidylglycine monooxygenase	6	2	39	8.18	22.78	0.03
21	K7WMX6	Venom peptide HsVx1	10	2	10.3	7.84	20.42	0.04
22	H2CYS4	Elastase-like protein (Fragment)	7	1	20.9	6.05	52.68	0.04
23	C5J889	Venom protein (Fragment)	19	2	12.9	6.55	186.99	0.05
24	P60253	Opicalcin-2	14	2	7.6	8.60	26.39	0.07
25	T1E6X4	CAP-Uro-1	4	1	38.7	6.42	297.38	0.07
26	P0DJ34	Potassium channel toxin kappa-KTx 2.7	31	1	7.1	4.42	1725.69	0.11
27	A0A443R344	Low-density lipoprotein receptor-like protein	17	1	10.3	5.21	107.87	0.20
28	C6H100	Hemocyanin subunit 5a	12	6	71.9	6.44	108.50	0.26

**Table 2.** The most upregulated venom proteins (ranked by Sequest HT score) identified in L. mucronatus venom. Data derived from LC-MS/MS analysis of the tryptic peptides.

No	Accession	Description	Coverage [%]	# Peptides	MW [kDa]	calc. pI	Score sequest HT	Abundance ratio: *(L. mucronatus)/(H. laoticus)*
1	P0CI49	Neurotoxin beta-KTx 31.1	41	5	10.8	8.75	299.17	100.00
2	A0AAV6TWV6	Uncharacterized protein	11	6	70.7	6.25	276.71	100.00
3	Q9UAC8	Beta-toxin BmKAs1	18	1	9.8	8.38	129.42	100.00
4	A0A2I9LP21	Hyaluronidase	5	2	46.4	8.54	88.07	100.00
5	P0CI51	Neurotoxin LmNaTx19	21	2	10.4	8.18	81.93	100.00
6	A0A2I9LP22	Hemocyanin AA6 chain	11	5	72	5.77	76.49	100.00
7	D9U2A1	Neurotoxin LmNaTx17	19	2	9.7	8.85	76.47	100.00
8	P0CI58	Neurotoxin LmNaTx64.1	28	2	10.2	9.13	74.13	100.00
9	A0A1E1WWA7	Putative hemocyanin subunit (Fragment)	13	6	71.9	6.18	66.17	100.00
10	A0AAV6V6V1	Uncharacterized protein	4	3	73.4	6.27	65.04	100.00
11	P0DJK8	Beta-toxin Im-2	22	1	7.9	8.47	62.34	100.00
12	A0A1E1WVP7	Putative hemocyanin subunit	10	5	71.9	6.01	61.59	100.00
13	A0A0U1TXP5	Neurotoxin LmNaTx4	12	2	9.4	8.51	53.74	100.00
14	A0A0U1SJ71	Neurotoxin LmNaTx12	9	1	9.7	8.60	45.71	100.00
15	A0A0U1TZ19	LCN-type CS-alpha/beta domain-containing protein	12	2	9.2	8.37	41.90	100.00
16	A0A0U1SN99	Neurotoxin LmNaTx18	13	1	9.8	8.84	31.70	100.00
17	P0CI60	Neurotoxin LmNaTx34.5 (Fragment)	13	1	9.5	7.50	27.35	100.00
18	A0A0U1S886	lysozyme	21	2	16.4	7.47	25.88	100.00
19	G8YZR4	Hemocyanin subunit d	6	3	72	6.24	32.24	94.48
20	D9U2A8	Potassium channel toxin alpha-KTx 15.9	15	1	6.5	8.50	26.89	54.84
21	A0A1E1WVS7	Putative hemocyanin subunit	17	11	72.7	5.83	123.90	46.12
22	P0CI42	Neurotoxin beta-KTx 14.3	12	2	9.4	7.99	44.75	42.07
23	A0A1E1WW95	Putative hemocyanin subunit	15	7	72.1	6.33	238.71	36.40
24	A0A1W7RAE6	Hemocyanin subunit 6	8	4	72.1	5.81	59.81	27.16
25	T1DPC1	CAP-Lyc-1	12	4	47	7.53	135.14	22.62
26	C5J8B0	Hemocyanin-like peptide (Fragment)	18	2	20.5	5.30	45.79	18.82
27	A0A1E1WVN1	Putative hemocyanin subunit	17	10	72.2	6.55	140.65	15.72
28	A0A1E1WWC5	Putative hemocyanin subunit	21	11	71.5	6.19	177.33	15.37
29	A0A1E1WVL6	Putative hemocyanin subunit	10	5	73.2	6.71	81.90	15.30
30	C6H0Z9	Hemocyanin subunit 6	11	6	71.7	6.10	50.80	12.08
31	C6H0Z6	Hemocyanin subunit 2 (Fragment)	12	5	69	5.88	41.63	9.61
32	Q3LGW2	Actin	24	7	41.8	5.48	79.62	3.44
33	A0AAQ4D3D3	Elongation factor 1-alpha	8	4	50.7	9.03	23.54	3.43
34	A0A8X6UFB5	Heat shock 70 kDa protein cognate 4	17	9	70.9	5.48	63.70	3.07
35	A0A8X6KIA3	Ras-related protein Rab-3	14	3	27.8	6.79	23.56	2.50
36	C6H102	Hemocyanin subunit 5b	10	5	73.7	6.19	82.23	1.72
37	P80476	Hemocyanin AA6 chain	10	5	71.7	5.82	41.58	1.00

### Effects of H. laoticus and L. mucronatus venoms on cardiovascular function 

**Figure 2.**
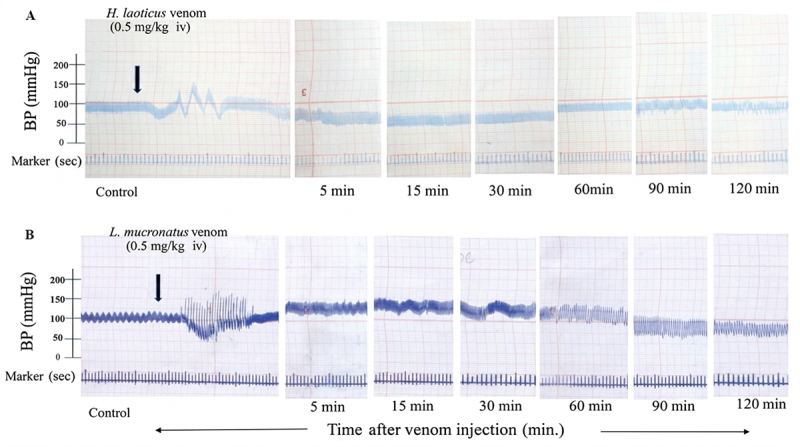
Typical effects of **(A)** H. laoticus and **(B)** L. mucronatus venoms on blood pressure (BP) and heart rate. The original tracings show arterial blood pressure responses (mmHg) following intravenous administration of venom (0.5 mg/kg), as indicated by the vertical arrow. Each trace represents data from a single rabbit receiving a single dose of venom (representative of four rabbits). The horizontal lines indicate the time course of the experiment. Each tracing displays blood pressure (mmHg) with corresponding time markers (sec).

Administration of H. laoticus venom (sublethal dose, 0.5 mg/kg BW, i.v.) produced an immediate depressor response in mean blood pressure (MBP), reaching a maximal decrease within 15 min (78.8 ± 3.5 mmHg vs. 96.3 ± 5.4 mmHg in the control, p < 0.05) (Table 3, Figure 2A). This transient reduction was followed by a gradual recovery toward pretreatment levels, and subsequently by a progressive terminal hypotensive phase between 30 and 120 min. In contrast, administration of L. mucronatus venom induced an initial rise in MBP (Figure 2B). within 5 min (111.6 ± 6.8 mmHg vs. 96.7 ± 5.8 mmHg in the control, p < 0.05), which persisted up to 15 min before declining below baseline at 30 min. MBP remained significantly lower than pretreatment values throughout the remaining experimental period at 60, 90, and 120 min (p < 0.05) (Table 3). These findings indicate species-specific differences in compensatory cardiovascular responses to venom-induced alterations in MBP. 

**Table 3.** Comparative effects of intravenous administration of H. laoticus and L. mucronatus venoms on heart rate (HR) and mean blood pressure (MBP). Data are expressed as mean ± SEM (n = 4).

	Time post-envenomation						
Parameters	Control	5 min	15 min	30 min	60 min	90 min	120 min
Heterometrus laoticus							
HR (beats/min)	273 ± 20	212 ± 29	250 ± 45	254 ± 49	272 ± 47	253 ± 37	233 ± 28
MBP (mmHg)	96.3 ± 5.4	76.7 ± 4.7*	78.8 ± 3.5*	81.7±4.5	84.6 ± 1.8	87.9 ± 4.1	89.6 ± 6.3
Lychas mucronatus							
HR (beats/min)	240 ± 21	246 ± 21	227 ± 26	173 ± 48	192 ± 46	174 ± 39	160 ± 34
MBP (mmHg)	96.7 ± 5.8	111.6 ± 6.8*	103.8 ± 6.9	88.0 ± 7.5	84.2 ± 5.4*	80.8 ± 5.1*	80.8 ± 5.3*

*Data were analyzed using repeated measures ANOVA with Bonferroni post-hoc test to determine significant differences (p < 0.05), comparing the specified time point to the internal control within the same group.

In the H. laoticus group, despite the reduction in MBP, heart rate (HR) remained unchanged throughout the 120 min observation period, suggesting that direct cardiac depression was unlikely to account for the acute hypotensive effect (Figure 2A). In contrast, in the L. mucronatus group, the initial elevation in MBP within 15 min was accompanied by a gradual decrease in HR starting at 30 min and persisting through 60, 90, and 120 min. These reductions in HR corresponded with sustained decreases in MBP relative to pretreatment values (Table 3).

### Effects of H. laoticus and L. mucronatus venoms on hematological profiles 

Comparative hematological responses to H. laoticus and L. mucronatus venoms are presented in Table 4. Red blood cell (RBC) counts tended to increase after envenomation in both groups, with significant elevations observed at 60 minutes (5.13 vs. 4.53 × 10⁶/μL of the control value, p < 0.05) and (5.32 vs. 4.97 × 10⁶/μL of the control value, p < 0.05) following H. laoticus and L. mucronatus venom administration, respectively. Hemoglobin (Hb) levels also showed significant increases at 30 and 60 minutes after envenomation with either H. laoticus venom (10.9 and 11.0 vs. 9.9 g/dL of the control value, p < 0.05) or L. mucronatus venom (11.8 and 11.9 vs. 10.0 g/dL of the control value, p < 0.05). Administration of H. laoticus venom induced a significant increase in mean corpuscular volume (MCV), from a control value of 65.7 to 72.9 fL within 5 minutes of envenomation (p < 0.05), whereas no significant change in MCV was observed after L. mucronatus venom administration. Hematocrit (Hct) levels were elevated throughout the experimental period in both groups, with significant increases within the first 15 minutes in the H. laoticus group (34.3 vs. 29.8% of the control value, p < 0.05) and within the first 30 minutes in the L. mucronatus group (34.1 vs. 29.9% of the control value, p < 0.05). In contrast, mean corpuscular hemoglobin (MCH) and mean corpuscular hemoglobin concentration (MCHC) showed no significant changes following administration of either venom. Notably, H. laoticus venom administration caused a marked decrease in platelet counts throughout the 60-minute observation period, with a significant reduction occurring within 5 minutes (152.5 vs. 246.0 × 10³/μL of the control value, p < 0.05). In contrast, no significant alterations in platelet counts were observed after L. mucronatus venom administration.

**Table 4.** Effects of H. laoticus and L. mucronatus venom on the hematological parameters of experimental rabbits at 5, 15, 30, 60, 90 and 120 minutes after venom administration.

Time post-envenomation							
Venom	Control	5 min	15 min	30 min	60 min	90 min	120 min
WBC (10^3^/ μL)							
H. laoticus	2.8 ± 0.6	2.7 ± 0.6	3.1 ± 0.4	3.5 ± 0.4	3.7 ± 0.3	4.2 ± 0.7*	4.4 ± 0.9*
L. mucronatus	3.7 ± 0.8	4.3 ± 1.0	5.4 ± 1.0	4.6 ± 0.7	4.5 ± 1.2	4.0 ± 1.1	3.9 ± 1.3
Neutrophil (%)							
H. laoticus	15.0 ±5.5	16.3 ± 1.6	11.3 ± 4.3	11.5 ± 3.6	13.2 ± 6.3	20.6 ± 10.5	13.1 ± 6.1
L. mucronatus	14.7 ±7.5	13.1 ± 7.7	18.5 ± 9.7	18.5 ± 11.6	22.2 ± 15.6	24.1 ± 13.7	22.6 ± 13.6
Lymphocyte (%)							
H. laoticus	80.2 ±5.3	90.5 ± 2.0*	85.3 ± 4.6*	84.7 ± 4.5*	82.2 ± 8.4	74.2 ± 12.5	74.9 ± 15.7
L. mucronatus	79.7 ±7.3	84.6 ± 8.1	79.2 ± 10.2	79.5 ± 12.4	75.3 ± 15.7	73.6 ± 14.7	72.2 ± 15.6
Monocyte (%)							
H. laoticus	2.2 ± 0.6	1.2 ± 0.2*	0.9 ± 0.3*	0.8 ± 0.1*	0.9 ± 0.2*	1.0 ± 0.2*	1.3 ± 0.4*
L. mucronatus	2.8 ± 0.9	0.8 ± 0.5	0.8 ± 0.4	0.9 ± 0.6	1.2 ± 0.7	0.9 ± 0.7	1.9 ± 2.2
Eosinophil (%)							
H. laoticus	1.2 ± 0.2	1.1 ± 0.6	1.4 ± 0.8	2.0 ± 1.0	2.7 ± 2.1	3.1 ± 2.4	4.7 ± 3.7
L. mucronatus	0.6 ± 0.1	0.4 ± 0.1	0.5 ± 0.2	0.4 ± 0.3	0.6 ± 0.4	0.5 ± 0.3	1.4 ± 1.1
Basophil (%)							
H. laoticus	1.4 ± 0.3	0.9 ± 0.3	1.1 ± 0.2	1.0 ± 0.3	1.0 ± 0.3	1.0 ± 0.3	1.30 ± 0.4
L. mucronatus	1.5 ± 0.8	1.1 ± 0.2	0.9 ± 0.2	0.7 ± 0.1	0.6 ± 0.2	0.9 ± 0.3	0.8 ± 0.2
RBC (10^6^/μL)							
H. laoticus	4.5 ± 0.1	4.7 ± 0.2	5.1 ± 0.2*	5.0 ± 0.1*	5.1 ± 0.2*	4.9 ± 0.3	4.7 ± 0.2
L. mucronatus	4.9 ± 0.3	5.2 ± 0.3	5.4 ± 0.3*	5.1 ± 0.3	5.3 ± 0.5*	5.0 ± 0.2	5.0 ± 0.4
HGB (g/dL)							
H. laoticus	9.9 ± 0.5	10.3 ± 0.6	10.9 ± 0.6*	11.0 ± 0.6*	11.2 ± 0.6	10.8 ± 0.7	10.5 ± 0.5
L. mucronatus	10.1± 0.5	11.5 ± 0.7	11.8 ± 0.7*	11.9 ± 0.8*	11.9 ± 1.3	11.4 ± 0.7	11.1 ± 0.9
HCT (%)							
H. laoticus	29.8 ± 1.9	33.9 ± 1.9*	34.3 ± 1.8*	33.4 ± 2.1	34.3 ± 2.5	33.1 ± 2.5	31.5 ± 1.9
L. mucronatus	29.9 ± 1.9	33.9 ± 1.8*	35.2 ± 1.9*	34.1 ± 2.0*	35.3 ± 3.5	34.2 ± 1.8	33.1 ± 2.5
MCV (fL)							
H. laoticus	65.7 ± 3.3	72.9 ± 4.3*	67.9 ± 3.6	66.9 ± 3.9	66.9 ± 4.0	56.9 ± 6.2	66.7 ± 3.9
L. mucronatus	64.8 ± 1.3	65.8 ± 0.8	65.8 ± 1.0	66.9 ± 0.9	66.4 ± 0.6	67.2 ± 0.8	66.7 ± 0.7
MCH (pg)							
H. laoticus	22.0 ± 0.7	22.1 ± 0.8	21.6 ± 0.9	22.0 ± 0.8	21.8 ± 0.8	21.8 ± 0.9	22.1 ± 0.9
L. mucronatus	22.4 ± 0.3	22.3 ± 0.2	22.0 ± 0.4	22.7 ± 0.2	22.4 ± 0.2	22.3 ± 0.4	22.4 ± 0.3
MCHC (g/dL)							
H. laoticus	33.5 ± 0.5	30.5 ± 1.4	31.9 ± 0.5	32.9 ± 0.6	32.6 ± 0.8	32.7 ± 0.5	33.2 ± 0.8
L. mucronatus	34.1 ± 0.4	33.9 ± 0.3	33.5 ± 0.2	34.0 ± 0.4	33.8 ± 0.4	33.2 ± 0.5	33.5 ± 0.6
PLT (10^3^/ μL)							
H. laoticus	246.0 ± 44.5	152.5 ± 18.0*	183.7 ± 25.3	205.5 ± 37.5	220.2 ± 46.4	263.0 ± 51.3	293.2 ± 46.1
L.mucronatus	375.5 ± 63.4	330.7 ± 42.8	348.0 ± 45.6	284.0 ± 29.5	327.6 ± 28.9	318.6 ± 9.3	277.6 ± 29.4
BUN (mg/dL)							
H. laoticus	16.4 ± 2.2	17.1 ± 2.2	16.6 ± 1.9	16.0 ± 2.0	15.1 ± 1.9	14.5 ± 2.1	14.3 ± 1.9
L. mucronatus	18.9 ± 2.4	19.5 ± 2.2	19.5 ± 4.8	18.6 ± 2.5	18.9 ±2.8	18.2 ± 2.3	18.5 ± 2.6
Creatinine (mg/dL)							
H. laoticus	0.8 ± 0.1	0.8 ± 0.1	0.7 ± 0.1	0.8 ± 0.1	0.7 ± 0.1	0.8 ± 0.1	0.7 ± 0.1
L. mucronatus	0.9 ± 0.1	0.9 ± 0.1	1.0 ± 0.1	0.9 ± 0.0	0.9 ± 0.0	0.8 ± 0.1	0.8 ± 0.1

WBC: white blood cell, RBC: red blood cell, HGB: hemoglobin, HCT: hematocrit, MCV: mean corpuscular volume, MCH: mean corpuscular hemoglobin, MCHC: mean corpuscular hemoglobin concentration, PLT: platelets. mean ± SEM, n = 4. *Data were analyzed using repeated measures ANOVA with Bonferroni post-hoc test to determine significant differences (p < 0.05) between the specified time point and the internal control within the same group.

Total white blood cell (WBC) counts tended to increase following administration of either H. laoticus or L. mucronatus venom, although the differential WBC profiles showed variable responses between the two groups. Neutrophil percentages consistently increased throughout the observation period, specifically at 60 and 120 minutes (22.2 ± 15.6% and 22.6 ± 13.6% vs. 14.7 ± 7.4% of the control value, p > 0.05) after L. mucronatus venom administration, whereas no significant changes were observed after H. laoticus venom administration. Lymphocyte percentages significantly increased at 5, 15, and 30 minutes (90.4%, 85.3%, and 84.7%, respectively, compared to 80.1% of the control value; p < 0.05) following H. laoticus venom administration, while no significant changes were evident after L. mucronatus venom administration. Monocyte percentages decreased in both groups after venom administration; however, the H. laoticus-treated group showed significant reductions in monocyte percentages, nearly one-fold lower than the control value (p < 0.05) throughout the experimental period. In addition, only the H. laoticus-treated group exhibited a nonsignificant elevation in eosinophil percentages throughout the study. No significant alterations were observed in basophil counts during the study period after administration of either venom.

The mean values of blood chemistry parameters, including blood urea nitrogen (BUN) and plasma creatinine concentrations, were not significantly affected by administration of either H. laoticus or L. mucronatus venom throughout the experimental period.

### Effects of administration of H. laoticus or L. mucronatus venom on renal hemodynamics 

**Figure 3.**
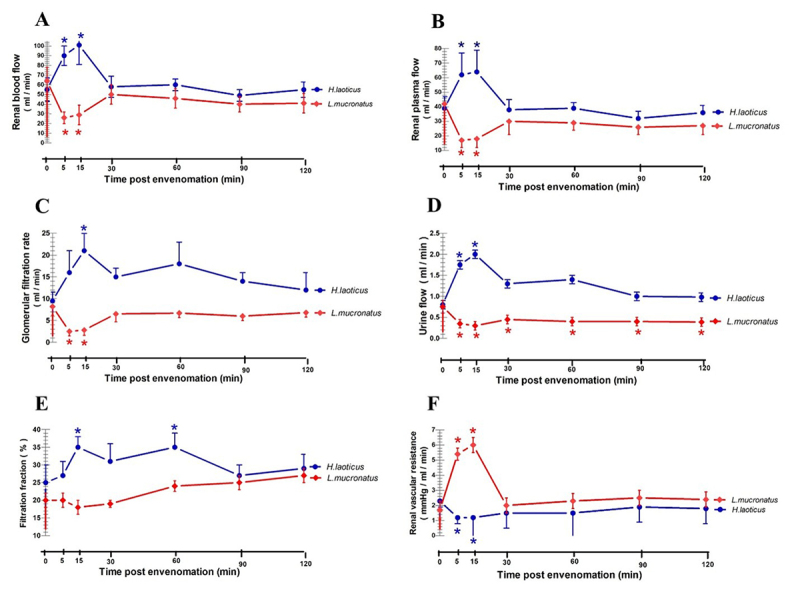
Comparative effects of administration of H. laoticus and L. mucronatus venom on the changes of renal hemodynamics: **(A)** renal blood flow, **(B)** effective renal plasma flow, **(C)** glomerular filtration rate, **(D)** urine flow, **(E)** filtration fraction, **(F)** renal vascular resistance. Mean ± SEM, n = 4. *Data were analyzed using repeated measures ANOVA with Bonferroni post-hoc test to determine significant differences (p < 0.05) between the specified time point and the internal control within the same group. *Data were analyzed using repeated measures ANOVA with Bonferroni post-hoc test to determine significant differences (p < 0.05) between the specified time point and the internal control within the same group.

Renal hemodynamic responses to H. laoticus and L. mucronatus venom administration are shown in Figure 3. Administration of L. mucronatus venom resulted in an immediate decrease in both renal blood flow (RBF) and effective renal plasma flow (ERPF) (Figures 3A and 3B). These decreases reached a maximal reduction 15 minutes after envenomation (29.0 vs. 64.9 mL/min of the control value for RBF, and 17.3 vs. 45.9 mL/min of the control value for ERPF, p < 0.05), followed by a gradual recovery to control levels. Renal vascular resistance (RVR) increased approximately threefold relative to the control value within 15 minutes (p < 0.05), followed by a gradual decline (30-120 min) to control levels after envenomation (Figure 3F). Administration of L. mucronatus venom also caused immediate decreases in glomerular filtration rate (GFR) and urine flow (UF), reaching their maxima 15 minutes after envenomation (2.8 vs. 8.3 mL/min of the control value for GFR, and 0.27 vs. 0.75 mL/min of the control value for UF, p < 0.05). These reductions were followed by a gradual recovery toward control levels (Figures 3C and 3D).

In contrast, H. laoticus venom produced the opposite effect, causing immediate and significant increases in RBF and ERPF, reaching maximal levels within 15 minutes after envenomation (101.0 vs. 56.0 mL/min of the control value for RBF, and 64.6 vs. 38.7 mL/min of the control value for ERPF, p < 0.05), followed by gradual recovery to control levels (Figures 3A and 3B). RVR decreased to approximately 48% of the control value at 15 minutes after H. laoticus venom administration (P < 0.05), followed by a tendency to decline throughout the experimental period. After the initial increases in both RBF and ERPF in animals treated with H. laoticus venom, GFR and UF also significantly increased, reaching their maxima at 15 minutes after envenomation (21.9 vs. 9.8 mL/min of the control value for GFR, and 0.20 vs. 0.81 mL/min of the control value for UF, p < 0.05). These increases were followed by a gradual return toward control levels (Figures 3C and 3D).

Comparative analysis of the filtration fraction (FF) between animals treated with H. laoticus and L. mucronatus venoms showed significant increases (p < 0.05) at 15 minutes (37.5 vs. 25.1% of the control value) and at 60 minutes (43.5 vs. 25.1% of the control value) after envenomation with H. laoticus venom, whereas administration of L. mucronatus venom showed no alteration in FF throughout the experimental period (Figure 3E).

*Effects of H. laoticus and L. mucronatus venoms on plasma electrolyte concentrations, fractional excretion of electrolytes, and osmolar clearance*

**Figure 4.**
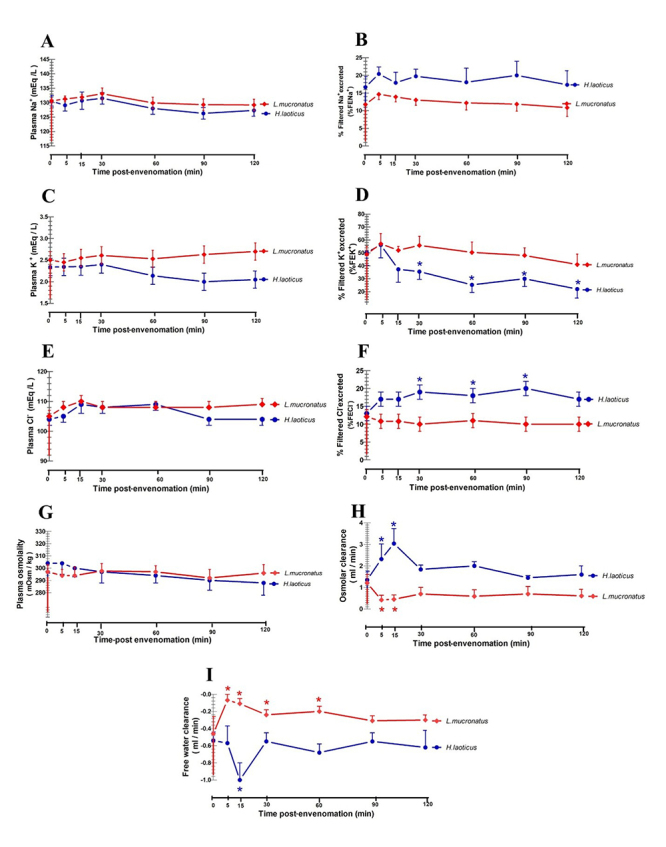
Comparative effects of intravenous administration of H. laoticus and L. mucronatus venoms on plasma electrolyte levels and renal electrolyte excretion. Panels show: **(A)** plasma Na⁺ concentration, **(B)** fractional Na⁺ excretion (%FE_Na⁺_), **(C)** plasma K⁺ concentration, **(D)** fractional K⁺ excretion (%FE_K⁺_), **(E)** plasma Cl⁻ concentration, **(F)** fractional Cl⁻ excretion (%FE_Cl⁻_), **(G)** plasma osmolality (P_osm_), **(H)** osmolar clearance (C_osm_), and **(I)** free water clearance (C_H₂O_). Values are expressed as mean ± SEM (n = 4). *Data were analyzed using repeated measures ANOVA with Bonferroni post-hoc test to determine significant differences (p < 0.05) between the specified time point and the internal control within the same group.

Administration of either H. laoticus or L. mucronatus venom did not produce significant changes in plasma sodium (P_Na⁺_), potassium (P_K⁺_), chloride (P_Cl⁻_), or plasma osmolality (P_osm_) compared with pretreatment values throughout the study period (Figures 4A, 4C, 4E, 4G). The fractional excretion of sodium (%FE_Na⁺_) showed no notable increase following administration of either venom (Figure 4B). H. laoticus venom induced significant decreases in urinary fractional excretion of potassium (FE_K⁺_), with reductions of 35%, 25%, 30%, and 22% relative to the control value (50%) at 30, 60, 90, and 120 min post-envenomation, respectively (p < 0.05). In contrast, L. mucronatus venom did not alter FE_K⁺_ throughout the experimental period (Figure 4D). Urinary fractional excretion of chloride (FE_Cl⁻_) remained unchanged following L. mucronatus venom administration, whereas H. laoticus venom caused significant increases (p < 0.05), with FECl⁻ values of 19%, 18%, and 20% at 30, 60, and 90 min, respectively, compared with 13% in controls (Figure 4F). Solute clearance (C_osm_) increased significantly immediately after H. laoticus venom administration, rising from 1.3 ml/min in controls to 2.3 ml/min and 3.0 ml/min at 5 and 15 min, respectively (p < 0.05; Figure 4H). Conversely, L. mucronatus venom caused a transient reduction in C_osm_ within the first 15 min (p < 0.05), followed by a gradual return to baseline values.

Free water clearance (C_H₂O_) increased significantly throughout the experimental period. Following L. mucronatus venom administration, this increase became significant within 60 min (p < 0.05), whereas H. laoticus venom caused a transient reduction in CH₂O during the first 15 min (p < 0.05) (Figure 4I). 

## Discussion

### Comparative effects of H. laoticus and L. mucronatus venoms on blood pressure and heart rate in rabbits 

The present findings indicate that the initial hypertensive effect induced by L. mucronatus venom is primarily mediated by its neurotoxic components. SDS-PAGE analysis of whole venom (Figure 1) together with LC-MS/MS profiling of its individual fractions revealed a high abundance of neurotoxins, particularly potassium channel toxins (β-KTx) and sodium channel toxins (NaTx). These proteins exhibit low molecular weights (9.4-10.8 kDa; Table 2), consistent with reported ion-channel-targeting neurotoxins in mammalian, crustacean, and insects [15, 16, 17]. The abundance and structural properties of these neurotoxins suggest a key role in the immediate cardiovascular responses following envenomation. β-KTx interferes with membrane repolarization in excitable cells [18], leading to increased sympathetic activity and transient vasoconstriction, which likely accounts for the rapid elevation in arterial blood pressure observed within the first few minutes after envenomation. Similarly, sodium channel toxins may delay the inactivation of voltage-gated sodium channels enhancing neuronal excitability, which can further stimulate catecholamine release from autonomic nerve terminals and the adrenal medulla, amplifying the hypertensive response [19]. Previous studies have defined the molecular mechanisms of action of scorpion neurotoxins as their interaction with ion channels in excitable cells, affecting ion permeation and voltage-dependent gating, which leads to massive neurotransmitter release [20]. Voltage-gated Na⁺ channel toxins are mainly responsible for the toxic and hypertensive effects of scorpion envenomation [21, 22].

Additionally, proteomic profiling indicates that the venom contains multiple isoforms of these neurotoxins, which may act synergistically to intensify their physiological effects. The small molecular size of these proteins facilitates rapid diffusion into the circulation, allowing them to reach target tissues such as smooth vascular muscle and cardiac tissue quickly, thereby producing the observed acute cardiovascular changes. Overall, the combined action of β-KTx and NaTx fractions, along with their high abundance in the venom, provides a mechanistic explanation for the prominent early-phase hypertension observed following L. mucronatus envenomation. However, the magnitude of the hypertensive response observed in this study was less pronounced than that typically seen in snake envenomation, such as that induced by Russell’s viper (Daboia russelii siamensis) [23]. It is likely that the initial significant increase in arterial blood pressure following L. mucronatus envenomation would be related to the direct action of its neurotoxic fractions on excitable membranes, resulting in the release of vasoconstrictor substances (e.g., catecholamines, renin-angiotensin, and/or endothelin) into the circulation. These mediators have been implicated in increasing total peripheral resistance and inducing hypertension, accompanied by a slight decrease in HR [24, 25, 26].

In the later phase, rabbits envenomed with L. mucronatus venom exhibited a prominent and long-lasting hypotensive response following the venom-induced hypertensive phase. The mechanism underlying this late-phase hypotension remains controversial. Proposed explanations include cholinergic effects, catecholamine depletion syndrome [27, 28], exaggerated β₂-mediated vasodilatory effects of circulating catecholamines on peripheral vessels, hypovolemia due to fluid loss, or the presence of potent vasodilatory substances such as kinins, cytokines, nitric oxide, and prostaglandins [26, 29, 30, 31, 32]. 

Regulation of blood pressure is usually mediated by coordinated interactions among various organs and systems, including the heart, vascular system, and central nervous control mechanisms. Therefore, the observed decrease in HR after L. mucronatus venom administration likely reflects the complex interplay among vascular control, baroreceptor reflexes, cardiovascular centers in the medulla, and changes in blood biochemistry. However, the precise mechanisms underlying these responses require further investigation.

In contrast to the hypertensive effect of L. mucronatus venom, administration of H. laoticus venom produced an initial decrease in MBP within 15 minutes, followed by gradual recovery toward pretreated levels, during which MBP remained lower than the control value. However, HR did not show significant alterations at any time point during the experimental period. The mechanisms underlying the initial hypotensive effect of H. laoticus venom are most likely multifactorial, involving several processes. The initial fall in MBP after H. laoticus venom administration was unlikely due to a direct reduction in cardiac output, suggesting other pathways for acute hypotension. These alterations may result from direct action of H. laoticus venom on vascular endothelium involved in blood pressure regulation, or from indirect release of endogenous vasodilators. Mass spectrometry analysis revealed a high proportion of PLA₂ components in H. laoticus venom (Table 1, Figure 1). Thus, the initial decrease in MBP following intravenous injection of H. laoticus venom was most likely mediated by its proteolytic content of phospholipase A_2_ [21]. Nevertheless, similar transient hypotensive effects mediated by PLA₂ action have been reported in snake venoms [33, 34]. The hemodynamic effects of H. laoticus venom resemble those of snake venoms, as a previous study using Daboia siamensis venom in dogs which confirmed that PLA₂ components in snake venom induce hemodynamic changes with hypotension [35]. PLA₂ activity may play an important role in altering the cell membrane permeability of vascular smooth muscle (VSM) cells, contributing to vascular relaxation and hypotension [36]. In the present study, the PLA₂ fraction and its derived phospholipids from H. laoticus venom are suggested to act as mediators of the hypotensive response. Their effects likely involve the generation of vasodilatory substances and the activation of cyclooxygenase-dependent pathways [37, 38]. Further studies are warranted to determine whether the hypotensive effect of H. laoticus venom is also mediated by histamine release in vivo in rabbits, as scorpion venoms are known to induce mast cell degranulation and subsequent histamine release [39, 40], and histamine acting on endothelial H1 receptors promotes nitric oxide generation, vasodilation, and rapid hypotension [41]. 

Unlike H. laoticus venom, PLA₂ components were not abundant in L. mucronatus venom. The present study identified the neurotoxic fraction of L. mucronatus venom as responsible for its hypertensive effect. This fraction contains highly basic, low-molecular-weight (9.4-10.8 kDa) proteins previously shown to be neurotoxic to insects, crustaceans, and mammals [15, 16]. However, this effect was less pronounced compared to hypertensive responses observed in snake venom envenomation, such as that of Russell’s viper [23].

### Effects of H. laoticus and L. mucronatus venoms on hematological responses 

Hematological parameters provide sensitive indicators of early systemic responses to envenomation. In this study, both L. mucronatus and H. laoticus venoms increased hematocrit (Hct), hemoglobin (Hb) and red blood cell (RBC) counts. Mean corpuscular volume (MCV was unchanged after L. mucronatus venom but increased significantly within 5 minutes following H. laoticus envenomation, coinciding with the rise in RBC count. Mean corpuscular hemoglobin concentration (MCHC) did not increase in either group, despite elevated Hb levels, indicating that hemolysis was not a major contributor. Although transient hemolyzed plasma was observed shortly after H. laoticus venom administration (unpublished observation). MCHC values did not correlate with Hb elevations, suggesting that hemolysis was limited and exposure-dependent. An inverse relationship between MCV and MCHC, particularly in the H. laoticus group, may reflect increased erythrocyte membrane permeability due to the high PLA₂ content of this venom. The consistent elevation in Hct in both groups suggests activation of compensatory mechanisms, possibly mediated by catecholamine-induced splenic contraction and erythrocyte mobilization [25, 42]

Leukocytosis is a common response to a wide variety of conditions, including venom exposure. [43]. L. mucronatus venom markedly increased neutrophil percentages without affecting lymphocyte levels, consistent with previous reports [44]. This neutrophilia may result from enhanced cytokine release, including interleukin-8 (IL-8), a potent neutrophil chemoattractant and activator [45], thereby contributing to systemic inflammatory cytokines responses [8, 45]. Both venoms caused a pronounced reduction in circulating monocytes, suggesting immune cell redistribution during envenomation. Monocytopenia may reflect inflammatory activation or toxic effects, as monocytes are key regulators of immune homeostasis and differentiate into macrophages and dendritic cells during inflammatory responses [46].

Platelet counts declined significantly within 60 min after H. laoticus venom administration, whereas no change was observed following L. mucronatus venom. This thrombocytopenia is consistent with findings in Daboia siamensis envenomation [47] and may result from splenic sequestration, immune-mediated destruction, reduced hepatic thrombopoietin production, or increased platelet consumption associated with vascular injury [48]. The high PLA₂ content of H. laoticus venom may further contribute by hydrolyzing platelet membrane phospholipids, promoting platelet aggregation and subsequent depletion from the circulation [49]. Additional venom components may also modulate platelet function, warranting further investigation.

### Effects of either H. laoticus or L. mucronatus venom administration on renal functions 

To clarify the relationship between venom action and renal function, we examined the effects of a single intravenous injection of H. laoticus or L. mucronatus venom on renal hemodynamics and urinary electrolyte excretion in rabbits. Alterations in renal function closely paralleled the cardiovascular effects of both venoms. Administration of H. laoticus venom (0.5 mg/kg, i.v.) induced rapid increases in glomerular filtration rate (GFR), urine flow (UF), effective renal plasma flow (ERPF), and renal blood flow (RBF) within 15 min, followed by a gradual decline, although values remained slightly elevated throughout the study (Figure 3). In contrast, L. mucronatus venom produced marked reductions in GFR, UF, ERPF, and RBF within the first 15 min, which persisted for the duration of experiment. Despite these pronounced hemodynamic changes, no histological evidence of acute tubular necrosis was observed, likely due to the relatively low venom dose used. Thus, intravascular coagulation was unlikely to contribute under these conditions.

The divergent renal responses likely reflect differences in venom composition, duration of renal ischemia, venom dose, and host responses. The predominant phospholipase A₂ (PLA₂) component of H. laoticus venom is likely responsible for its systemic vasodilatory effects, as evidenced by decreased arterial pressure and renal vascular resistance (RVR) within 15 min. A disproportionate increase in GFR relative to ERPF resulted in an elevation of the filtration fraction. The hydrolytic activity of PLA₂ may disrupt glomerular basement membrane phospholipids, increase filtration barrier permeability and enhance inulin clearance, thereby elevating GFR and UF [50].

Conversely, L. mucronatus venom, which lacks abundant PLA₂, contains high levels of neurotoxins that likely induce renal vasoconstriction, reflected by a marked increase in RVR and sustained reductions in GFR, UF, RBF and ERPF. The present findings identify the neurotoxic fraction as the principal mediator of renal vasoconstriction, coinciding with the hypertensive response. Reduced GFR and UF are therefore attributed to decreased renal perfusion. Potential mechanisms include activation of the renin-angiotensin system during envenomation [51], local release of platelet-activating factor [52], and increased thromboxane B_2_ production, which promotes mesangial cell contraction and reduce the glomerular filtration surface area and ultrafiltration coefficient (Kf) [53, 54]. In addition, potassium channel toxins (KTx) abundant in L. mucronatus venom may cause afferent arteriolar constriction via smooth muscle depolarization, further contributing to reduced renal blood flow and filtration [55].

These results indicate that distinct venom fractions differentially modulate renal function, highlighting the importance of venom-specific mechanisms and their direct actions on renal tissues. Although renal ischemia secondary to renal vasoconstriction can impair renal function, neither H. laoticus nor L. mucronatus venom induced acute renal failure, as plasma urea and creatinine levels remained unchanged throughout the experiment. The kidney is a major route of venom elimination [56], and radioisotope studies in rats have shown rapid venom clearance from the circulation with substantial renal uptake, indicating relatively slow renal elimination. More pronounced renal hemodynamic changes may have been detected with longer observation periods.

Neither venom altered plasma Na⁺, K⁺, or Cl⁻ concentrations; however, H. laoticus venom tended to increase fractional Na⁺ and Cl⁻ excretion, consistent with an osmotic diuretic effect that contributed to increased urine output. In contrast, fractional K⁺ excretion was significantly reduced and remained suppressed throughout the experimental period. This reduction may be attributed to the abundance of potassium channel toxins (α-KTx and κ-KTx) in H. laoticus venom, which likely inhibit renal K⁺ channels and associated transporters, including NHE3, ENaC, and Na⁺/K⁺-ATPase, thereby limiting tubular K⁺ secretion [57]. Inhibition of NHE3 activity may further reduce K⁺-H⁺ exchange at the apical membrane, decreasing urinary K⁺ excretion [58].

Additionally, the high PLA₂ content of H. laoticus venom may disrupt tubular basement membrane integrity, leading to increasing paracellular Na⁺ leakage and the generation of an osmotic diuresis characterised by enhanced osmolar clearance, increased urine flow, and increased free water clearance. This mechanism provides a plausible explanation for the trends of increased %FENa⁺ and %FECl⁻ and decreased %FEK⁺ observed in this study (Figures. 4B, 4D, 4F). However, the lack of significant changes in %FENa⁺ suggests that Na⁺/K⁺-ATPase activity in the kidney was largely preserved under the present experimental conditions.

In summary, abundant potassium channel toxins in H. laoticus venom likely inhibit renal K⁺ channels, whereas KTx-family neurotoxins in L. mucronatus venom may induce vasoconstriction and hypertension. Both effects may involve aldosterone-mediated regulation of serum K⁺ during envenomation [59], although hormonal response involving aldosterone was not assessed in this study, representing a limitation.

However, this study has several limitations. The relatively low venom dose and short observation period, while suitable for preliminary analysis, may limit the generalizability of the findings and fail to capture the full spectrum of cardiovascular and renal pathophysiology. In addition, the specific actions of individual venom fractions on blood pressure and renal function were not fully characterized. Future studies should focus on defining the organ-specific effects of isolated venom components, their roles in inducing vasoactive mediators, and their temporal effects over longer observation periods. The potential for venom components to act independently or synergistically, as well as the contribution of inflammatory and oxidative stress pathways, also warrants further investigation.

## Conclusions

The present study demonstrates that scorpion venoms from two Southeast Asian species elicit distinct cardiovascular and renal pathophysiological responses in rabbits. These acute effects are mediated by specific venom components, including K⁺ and Na⁺ channel neurotoxins and phospholipase A₂. The high abundance of KTx and NaTx in L. mucronatus venom likely underlies its pronounced neurotoxic effects, causing initial vasoconstriction, hypertension, and reduced renal hemodynamics. In contrast, the PLA₂-rich venom of H. laoticus appears to drive cytotoxic mechanisms that induce vasodilation, hypotension, and enhanced renal hemodynamics. These findings provide mechanistic insight that may inform improved strategies for the management of scorpion envenomation.

### Abbreviations

2D-GE: two-dimensional gel electrophoresis; C: clearance; C_in_: inulin clearance; C_PAH_: PAH clearance; C_H₂O_: free water clearance; C_osm_: osmolar clearance; ENaC: epithelial sodium channels; ERPF: effective renal plasma flow; FE_Cl-_: fractional chloride excretion_;_ FE_K+_: fractional potassium excretion; FE_Na+_: fractional sodium excretion; FF: filtration fraction; GFR: glomerular filtration rate; HCT: hematocrit; HGB: hemoglobin; HR: heart rate; H. laoticus: Heterometrus laoticus; In: inulin; i.v.: intravenous injection; Kf: ultrafiltration coefficient; KTx: potassium channel toxins; LC-MS/MS: liquid chromatography tandem mass spectrometry; L. mucronatus: Lychas mucronatus; MBP: mean arterial blood pressure; MCV: mean corpuscular volume; MCH: mean corpuscular hemoglobin; MCHC: mean corpuscular hemoglobin concentration; MS: mass spectrometry; NaTx: sodium channel toxins; NHE3: sodium-hydrogen exchanger 3; PAF: platelet-activating factor; PCV: packed cell volume; PLA_2_: phospholipase A_2_; PLT: platelets; RBC: red blood cells; RBF: renal blood flow; RVR: renal vascular resistance; SDS-PAGE: sodium dodecyl sulphate polyacrylamide gel electrophoresis; TxB2: thromboxane B2; UF: urine flow rate; WBC: white blood cell.

## Data Availability

All data generated and analyzed during this study are included in this published article.
